# Immunogenicity and Antigenicity of the Recombinant Ectodomain of Rabies Virus Glycoprotein Containing the Human Collagen XVIII Trimerization Domain

**DOI:** 10.3390/vaccines13090971

**Published:** 2025-09-12

**Authors:** Izat Smekenov, Gulshat Bayandy, Sanzhar Alybayev, Nuraiym Baltakhozha, Zhanat Batanova, Nurlan Akhmetsadykov, Amangeldy Bissenbaev

**Affiliations:** 1 Department of Molecular Biology and Genetics, Faculty of Biology and Biotechnology, Al-Farabi Kazakh National University, Almaty 050040, Kazakhstan; smekenovizat@gmail.com (I.S.); bayandy.gulshat92@gmail.com (G.B.); sanzhar.alybayev@gmail.com (S.A.); baltakhozhanuraiym@mail.ru (N.B.); 2 Scientific Research Institute of Biology and Biotechnology Problems, Al-Farabi Kazakh National University, Almaty 050040, Kazakhstan; 3 LLP Research and Production Enterprise “Antigen”, Almaty 040905, Kazakhstan; zhanat.batanova@gmail.com (Z.B.); nurlan.akhmetsadykov@gmail.com (N.A.)

**Keywords:** rabies, glycoprotein, expression, ELISA, mice immunization, trimeric protein, human collagen XVIII, immunogenicity

## Abstract

**Background**: Rabies remains a fatal zoonotic disease, necessitating effective and affordable vaccines. While current vaccines are effective, they require multiple doses and may not induce long-lasting immunity in all settings. The rabies virus glycoprotein (RABV-G) is the principal antigen responsible for eliciting virus-neutralizing antibodies, but its recombinant monomeric forms often suffer from poor immunogenicity due to misfolding and aggregation. **Methods**: A recombinant trimeric RABV-G ectodomain (rRABV-G-XVIII) was engineered by fusing it to a human collagen XVIII-derived trimerization domain. The protein was expressed in *E. coli*, purified under denaturing conditions, and refolded. Trimer formation was verified using size-exclusion chromatography. Mice were immunized with rRABV-G-XVIII, with or without adjuvant, and compared to a monomeric form (rRABV-GE). Antigen-specific antibody responses were measured by ELISA, neutralizing activity was assessed, and protective efficacy was evaluated via intracerebral challenge with the CVS-27 rabies strain. **Results**: rRABV-G-XVIII formed stable trimers and induced strong humoral immune responses, with high ELISA titers and virus-neutralizing activity comparable to an inactivated rabies vaccine. Mice immunized with rRABV-GE showed lower antibody responses and partial protection, which improved with adjuvant. All rRABV-G-XVIII-immunized mice were fully protected against rabies challenge, independent of adjuvant use. **Conclusions**: Stabilization of RABV-G in its native trimeric conformation markedly improves immunogenicity and protective efficacy. This approach offers a promising strategy for the development of rabies subunit vac-cines with simplified formulations and potential for cost-effective production in bacterial systems.

## 1. Introduction

The rabies virus (RABV) is a member of the *Lyssavirus* genus within the *Rhabdoviridae* family. This virus is the primary cause of rabies, a fatal neurological disease in mammals, including humans [1]. Globally, rabies is still endemic in over 150 countries, and it accounts for more than 60,000 human deaths annually (of which 96% of the cases occur in Asia and Africa) [2,3]. Productive infection of humans with any *Lyssavirus*, including rabies virus and Australian bat lyssavirus, is nearly always fatal once clinical signs develop [4]. While rabies can infect any mammal, it is most commonly found in wild and domestic canines, cats, skunks, raccoons, and bats. The virus is primarily transmitted through the saliva of an infected animal, usually via a bite or scratch, and can also be transmitted through mucous membranes or fresh wounds [5]. Rabies is a deadly yet preventable virus, with vaccines administered before or after exposure to a rabid animal.

Both human and animal rabies vaccines are available and are crucial for rabies control. These vaccines are used for both pre-exposure prophylaxis (PrEP), for those at high risk, and post-exposure prophylaxis (PEP), after a potential exposure. Most of the traditional rabies vaccines use complete, inactivated viruses with the same antigenic properties as wild-type viruses [6]. Despite rabies being preventable through vaccination, it remains a significant public health issue in countries where the rabies virus is endemic and poses a serious risk to travelers visiting these regions. Currently, the most effective strategy to control and prevent human rabies involves vaccinating wild, feral, and domestic carnivores. Pre-exposure immunization against rabies relies on multi-dose regimens of inactivated whole-virus vaccines, which, while effective, have drawbacks. Production requires high-biosafety-level labs for live virus growth, mandatory inactivation before vaccine batch release, and extensive safety testing using laboratory animals. Moreover, achieving sufficient antibody levels for protection necessitates multiple high-dose vaccinations [7]. For these reasons, efforts have been made to develop alternative vaccine production methods.

The native rabies G protein (RABV-G) is a 505-amino-acid glycoprotein that forms homotrimers through oligomerization, which is essential for interaction with target membrane receptors and for inducing neutralizing antibodies that confer protection against rabies infection [8,9]. For these reasons, most of the recombinant vaccine candidates described today are based on the RABV-G protein. Various expression systems, including *E. coli*, yeast cells, insect cells, mammalian cells, and viral vectors, have been utilized to express the RABV-G [10,11,12,13,14,15]. These recombinant proteins have been evaluated for their ability to induce neutralizing antibodies and protective immunity in animal models. Additionally, strategies involving the delivery of RABV-G through viral vectors (adenovirus and others) to elicit in vivo expression have been used [16,17]. Some of these candidates have been shown to be immunogenic in animals, but none have so far been registered for human or animal use.

In the present work, we generated a system for producing a protein subunit vaccine using an *E. coli*-derived rabies RABV-G ectodomain modified with a human collagen XVIII trimerization domain [18] to stabilize the native trimeric structure of the RABV-G ectodomain. We determined the effect of this stabilization on the immunogenicity of the protein vaccine and its ability to induce a protective immune response to challenge with rabies virus in a mouse model. Collectively, these data hold promise for the development of novel generations of effective, cheap, and safe vaccines against rabies.

## 2. Materials and Methods

### 2.1. Construction of Candidate Vaccines

A sequence encoding the RABV-G ectodomain (RABV-GE, amino acids [aa] 20 to 458) of RABV strain CVS-11 (GenBank accession number GQ918139.1) with collagen XVIII trimerization domain was synthesized (rRABV-G-XVIII, GeneCust Europe, Dudelange, Luxembourg). Before gene synthesis, the hydrophobic regions spanning residues 73–79 and 117–125 were substituted with a flexible GGSGG linker. Additionally, the sequence was optimized for codon usage and GC content to enhance expression in *E. coli* [19]. In addition, a flexible linker, AAANSGAGGSGGSSGSDGA [20,21], was placed between rRABV-GE and the collagen XVIII trimerization domain. This sequence from the pBSK II (+) vector was inserted into the *Nco*I and *Xho*I restriction sites of the pET-28c (+) vector, thereby adding 6× His-tag coding sequences to the 3′-end of the gene (pET-28c-rRABV-G-XVIII). The gene part encoding the extracellular domain of rRABV-GE, with a length of 433 amino acids, was derived from pET-28c-rRABV-G-XVIII using PCR and then cloned into the pET-28c (+) vector at the same restriction sites. All plasmids were sequenced to confirm deduced amino acid sequences. The resulting expression plasmids, pET-28c-rRABV-GE and pET-28c-rRABV-G-XVIII, yielded the respective proteins with a C-terminal 6xHis-tag sequence.

### 2.2. Recombinant Protein Expression and His-Tag Affinity Purification

The chemically competent *E. coli* Shuffle^®^ T7 Express LysY strain (C3030) (New England Biolabs Inc., Ipswich, MA, USA) was prepared and transformed with recombinant plasmids using the standard heat shock protocol [22,23]. Transformed cells were spread onto LB agar plates supplemented with 50 µg/mL kanamycin to select colonies that had successfully taken up the plasmids. Recombinant cells harboring pET-28c-rRABV-GE and pET-28c-rRABV-G-XVIII plasmids were screened on selective plates supplemented with kanamycin. A positive clone was chosen and grown for expression protein overnight in LB medium with 50 µg/mL kanamycin at 37 °C, shaking at 180 rpm. The overnight culture (10 mL) was inoculated into 500 mL LB medium supplemented with 50 µg/mL kanamycin and grown at 37 °C with shaking at 180 rpm. Then, 0.5 mM isopropyl-β-D-galactopyranoside (IPTG; Thermo Scientific™, Waltham, MA, USA) was added to the culture medium and incubated for 16 h at 30 °C after optical density at 600 nm (OD_600_) reached 0.6. After induction, the bacterial cells were harvested by centrifugation at 4000× *g* for 10 min at 4 °C, and the pellet was resuspended in lysis buffer (50 mM sodium phosphate buffer, pH 7.5, 300 mM NaCl, 1 mM EDTA pH 8.0, 1 mM DTT, 5 mM β-mercaptoethanol, 2% Triton X–100, 5% glycerol, 0.1 mM phenyl-methyl-sulfonyl fluoride (PMSF)), supplemented with protease inhibitor cocktail (PIC; Roche, Basel, Switzerland) and sonicated on ice using an Omni Ruptor 4000 (Omni International, Kennesaw, GA, USA) for 6 cycles of 30 s ON/90 s OFF. The sonicated lysate was then spun at 12,000× *g* for 30 min at 4 °C, and the resulting pellet, which contained inclusion bodies, was dissolved in refolding buffer (50 mM sodium phosphate, pH 7.5; 500 mM NaCl; 5 mM β-mercaptoethanol; 10 mM imidazole) supplemented with 8 M urea. The clear dialysate was loaded onto a nickel–nitrilotriacetic acid (Ni-NTA) affinity chromatography column 1 mL HisTrap HP (Cytiva, Uppsala, Sweden). Impurities were removed by washing the column with 15 column volumes (CV) of refolding buffer. Elution buffer (50 mM sodium phosphate buffer pH 7.5, 500 mM NaCl, 5 mM β-mercaptoethanol, 500 mM imidazole, 8 M urea) was used to elute the proteins. The protein elution fractions were monitored by measuring absorbance at 280 nm, and the fractions corresponding to the peaks were stored at −20 °C. The collected fractions containing the recombinant proteins were pooled and dialyzed stepwise against refolding buffer 2 (50 mM sodium phosphate buffer pH 7.5, 150 mM NaCl, 500 mM L-Arginine, 20% glycerol) containing 6 M, 4 M, 2 M, or 0 M urea. Purified proteins were resolved on a 10% SDS-PAGE gel, transferred to membranes, and probed either with a rabbit monoclonal antibody against the 6× His tag (ab200537; Abcam, Cambridge, UK) or with polyclonal sera from mice immunized with inactivated RABV CVS-11. Detection was performed using the Clarity™ Western ECL substrate (Bio-Rad, Hercules, CA, USA).

### 2.3. Analysis of Proteins by Size Exclusion Chromatography

To examine their oligomeric states, size exclusion chromatography (SEC) of rabies glycoprotein proteins (rRABV-GE and rRABV-G-XVIII) was performed on a pre-packed Superose 12 10/300 GL column (GE Healthcare, Uppsala, Sweden) in 50 mM phosphate buffer pH 7.5, 150 mM NaCl. Purified proteins were filtered with a 0.22 µm filter to remove possible insoluble protein aggregates prior to SEC. Then, 500 µL of the protein samples at a concentration of 2.0 mg/mL were loaded. Chromatography was performed at a flow rate of 0.4 mL per minute. The eluted proteins were detected with UV light at 280 nm and confirmed by SDS-PAGE. For comparison of the molecular mass of G proteins, the gel filtration column was calibrated with standard protein molecular mass markers.

### 2.4. Immunization and Challenge of Mice

Six groups of 6–8-week-old female BALB/C mice (six per group) were vaccinated via intramuscular inoculation. One group of mice was immunized twice with the inactivated rabies virus vaccine (CVS-11 strain) [24] at days 0 and 14. Another control group received 1× PBS alone on the same days. Mice receiving the adjuvanted recombinant protein were immunized intramuscularly with 30 µg of purified rRABV-GE or rRABV-G-XVIII protein containing 50% complete Freund’s adjuvant (Sigma, St. Louis, MO, USA). The immunized mice were later given booster doses at day 14, using the same amount of protein in 50% emulsion with Freund’s incomplete adjuvant (Sigma, St. Louis, MO, USA). The blood of mice was collected at fourteen days after the second immunization by an oblique superficial cut of the tail. Serum collected on day 0 before the administration of vaccines served as a pre-immune control. Mouse sera were individually collected and clarified by centrifugation. To deactivate non-specific complement activity, the sera were incubated at 56 °C for 30 min prior to storage at −20 °C.

Fourteen days after the second dose of vaccine, mice from both the vaccinated and control groups were anesthetized (Zoletil, Virbac, Carros, France) and intracerebrally challenged with 30 µL volumes containing 30 LD_50_ of CVS-27. The CVS-27 strain is well-established for reproducible intracerebral infection in mice and has been commonly used in mouse survival models [25]. The challenge dose was chosen based on preliminary experiments in our lab, which showed that this dose led to a 100% mortality of infected mice. Following the virus challenge, the mice were closely monitored until the end of the experiment on day 60. Control animals (PBS-immunized) developed characteristic clinical signs of rabies infection, including progressively disheveled fur, hunched posture, lethargy, and hind-leg paralysis and impaired mobility, within 5–8 days post-challenge. According to humane endpoint criteria, animals exhibiting severe neurological symptoms were euthanized and recorded as non-survivors. The animal experiments were approved by the Local Ethics Committee of the LLP Scientific and Production Enterprise “Antigen” (protocol code #14, 25 November 2024). All animals used in this research were cared for in accordance with “The Code for the Care and Use of Animals in Research”.

### 2.5. Enzyme-Linked Immunosorbent Assays

In an enzyme-linked immunosorbent assay (ELISA), 100 µL of purified rRABV-GE, rRABV-G-XVIII, or inactivated rabies virus CVS-11 strain in 1× PBS (137 mM NaCl, 2.7 mM KCl, 10 mM Na_2_HPO_4_, 1.8 mM KH_2_PO_4_, pH 7.4) at 1 µg/mL concentrations was coated onto 96-well ELISA plates Nunc MaxiSorp^®^ (Thermo Fisher Scientific, Waltham, MA, USA) and allowed to bind overnight at 4 °C with shaking at 600 rpm/min. Plates were then washed five times with washing buffer (PBST, 0.05% Tween-20 in 1× PBS) and blocked with 3% BSA in washing buffer at room temperature for 1 h. After washing three times with washing buffer, 100 µL of each serially diluted mouse serum for all mouse groups in 1% (*w*/*v*) BSA in PBST at ratios of 1:500, 1:1000, 1:2000, 1:4000, 1:8000, 1:16,000, 1:32,000, 1:64,000, 1:128,000, and 1:256,000 was then added to the plates and incubated at room temperature for 1 h. Plates were again washed five times with washing buffer, and then wells were incubated with goat anti-mouse IgG conjugated to horseradish peroxidase (1:5000 in 1× PBST) for 1 h at 37 °C, followed by five washes. TMB substrate (Thermo Fisher Scientific, Waltham, MA, USA) was added to each well, and color was allowed to develop. Color development was stopped using 1 M sulfuric acid. Absorbances were read at 450 nm on a microplate reader (DAS, Palombara Sabina, Rome, Italy). Endpoint titers were determined as the highest serum dilution yielding an OD450 ≥ 0.1.

### 2.6. Neutralization Assay

Vero cells (1.5 × 10^4^) were seeded in a 96-well plate (Corning Incorporated, Corning, NY, USA) with DMEM F-12 (Gibco, Grand Island, NY, USA) containing 5% FBS (Capricorn Scientific, Ebsdorfergrund, Germany) at 37 °C in a humidified atmosphere with 5% CO_2_ for 24 h. Serum samples from mice immunized with rRABV-GE and rRABV-G-XVIII protein vaccine with or without adjuvant were tested. All of the pooled sera were heat-inactivated at 56 °C for 30 min and then two-fold serially diluted with complete DMEM containing 10% FBS (Capricorn Scientific, Ebsdorfergrund, Germany). The sera were mixed with an equal volume of virus dilutions containing approximately 100 TCID_50_ of the CVS-11 strain rabies virus and incubated for 2 h at 37 °C. The serum–virus mixtures were pipetted into designated wells (from well 1:2 to 1:512). Positive controls, using serum from mice immunized with inactivated rabies virus vaccine, were included in the assay. Then, the plate was incubated at 37 °C with 5% CO_2_ for 6 days, after which the Vero cells were examined under an inverted light microscope for any cytopathic effect (CPE). Neutralization titers were determined as the highest dilutions that resulted in a 100% inhibition of CPE.

## 3. Results

### 3.1. Production of Recombinant RABV-G Proteins

**Figure 1:**
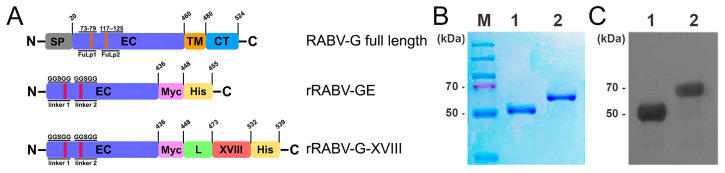
Schematic representation and SDS-PAGE analysis of recombinant soluble RABV-G constructs. (**A**) Recombinant soluble RABV-G constructs. Full-length RABV-G. SP—signal peptide, EC—ectodomain, TM—transmembrane domain, CT—cytosolic domain. The RABV-G ectodomain (rRABV-GE) construct was generated by eliminating the transmembrane (TM) and cytosolic (CT) domains. The modified trimeric RABV-G (rRABV-G-XVIII) was generated by fusing the collagen XVIII trimerization domain to the C-terminus of rRABV-GE with a short peptide linker. In rRABV-GE and rRABV-G-XVIII, hydrophobic residues at 73–79 and 117–125 were replaced with the Gly-Gly-Ser-Gly-Gly linker. SDS-PAGE (**B**) and Western blots (**C**) of affinity-purified rRABV-GE (lane 1) and rRABV-G-XVIII (lane 2) proteins. M—PageRuler™ Plus Prestained Protein Ladder, 10 to 250 kDa.

A cDNA-containing gene for the putative rabies G protein ectodomain (RABV-GE) and RABV-GE modified with human collagen XVIII trimerization domain (rRABV-G-XVIII) proteins were obtained as described in the “Materials and Methods” section. Diagrams of recombinant *E. coli* expression constructs for RABV-GE and rRABV-G-XVIII are shown in Figure 1A. The sequences of the putative fusion loops in the RABV-G extracellular domain in the regions between amino acids 73–79 and 117–125 are more abundant in hydrophobic amino acids, and this often causes the recombinant protein to aggregate in solution, which affects the homogeneity of the protein [19]. Putative fusion loops in the RABV-GE are small, about 17–19 amino acids, which is why a substitution of the hydrophobic amino acids to Gly-Gly-Ser-Gly-Gly linkers does not affect the folding or the antigenicity. The above fusion loop substitution strategy allows a soluble and properly folded RABV-GE extracellular domain to be obtained, as previously observed [19]. A flexible linker, GGSGG, was placed between rRABV-GE and the collagen XVIII trimerization domain. In addition, a C-terminal His-tag was introduced to facilitate affinity purification.

The rRABV-GE and rRABV-G-XVIII coding sequences were cloned in the expression vector pET28c (+) and, after transformation of the *E. coli* Shuffle^®^ T7 Express LysY strain (C3030), the expressed proteins, following 16 h incubation in the presence of IPTG, were monitored by SDS-PAGE. However, despite the replacement of the fusion loops, recombinant proteins were detected in the insoluble fraction of the cell extract. Since all recombinant protein was detected in the insoluble fraction of the cell extract, different refolding methods were tested in order to generate a soluble recombinant protein. The recombinant proteins, which were expressed as an inclusion body, were subsequently solubilized using 8 M urea; hence, purification of the recombinant proteins was carried out under denaturing conditions by nickel affinity chromatography. After purification as described in the “Materials and Methods” section, refolding was performed with descending denaturant concentration dialysis. Samples of each of the purified proteins were analyzed for purity using SDS-PAGE and Western blots (Figure 1B,C; lanes 1 and 2). Coomassie blue staining of SDS-PAGE revealed a single band for both rRABV-GE and rRABV-G-XVIII proteins, indicating their high purity (Figure 1B). The larger size of rRABV-G-XVIII suggests the addition of the collagen XVIII trimerization domain at its C-terminus. In addition, anti-His monoclonal antibody binding confirms the presence of the recombinant proteins (Figure 1C; lanes 1 and 2). The molecular weights were approximately 51 and 60 kDa for rRABV-GE and rRABV-G-XVIII, respectively. The final protein yield following the refolding and purification steps reached 3.5 mg of protein per liter of bacterial culture.

**Figure 2:**
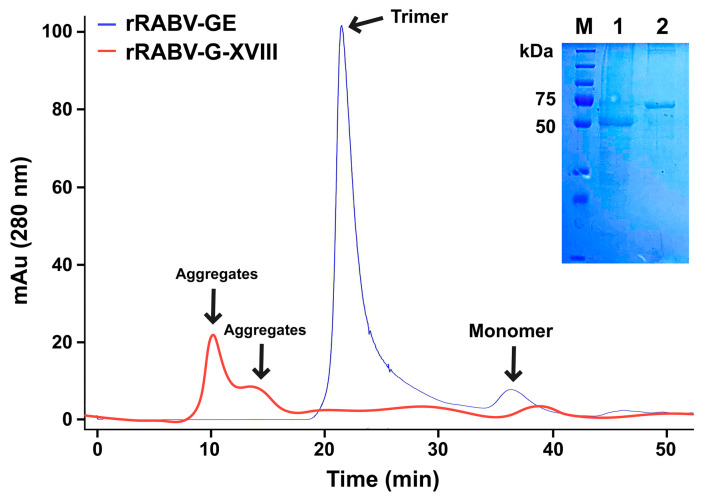
Characterization of the oligomeric state of rRABV-G-XVIII using size-exclusion chromatography. Fusion of the trimerization region of the C-terminal non-collagenous domain (NC1) of collagen XVIII to the C-terminus of rRABV-GE (rRABV-G-XVIII) results in the formation of a stable trimeric structure. Superose 12 10/300 GL gel filtration chromatography analysis of purified monomeric and trimeric proteins. The SDS-PAGE analyses of the eluted monomeric and trimeric proteins are shown. mAU—milli-absorbance units; M—PageRuler™ Plus Prestained Protein Ladder, 10 to 250 kDa; 1—eluted sample of the aggregate fraction; 2—eluted sample of the trimer fraction.

To examine their oligomeric states, the purity of rRABV-G-XVIII was analyzed by gel filtration chromatography using a Superose 12 10/300 GL column. The analysis showed a main peak at approximately 250 kDa, representing 96.3% trimeric protein, with a minor peak at around 61 kDa identified as monomeric protein (Figure 2). In contrast, rRABV-GE, lacking the trimerization domain, formed aggregates during gel filtration (Figure 2). SDS–PAGE analysis of the gel filtration fractions confirmed the presence of rRABV-G-XVIII in the main peak, showing a single band at 60 kDa, while the aggregate peak corresponded to the molecular mass of rRABV-GE (51 kDa).

### 3.2. In Vivo Humoral Immune Responses

**Figure 3:**
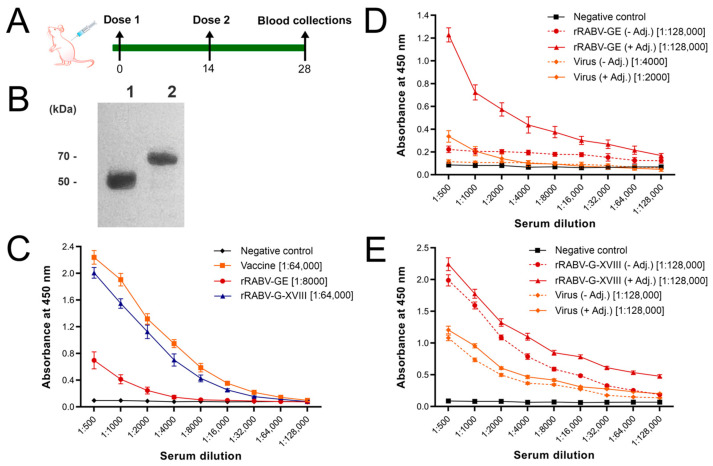
Immunization with trimeric rRABV-G-XVIII induces a robust and specific humoral immune response. (**A**) Immunization scheme of mice with rRABV-GE (ectodomain, 30 µg) and rRABV-G-XVIII (RABV-GE modified with a human collagen XVIII trimerization domain, 30 µg) with or without adjuvant. One group of mice was immunized twice with a commercially available RABV vaccine (inactivated CVS-11 virus). (**B**) Western blot analysis of rRABV-GE (lane 1) and rRABV-G-XVIII (lane 2) proteins using polyclonal sera from the inactivated rabies virus. (**C**) Antibody titer of anti-virus sera with rRABV-GE and rRABV-G-XVIII and purified virus in ELISA. (**D**) Antibody titer of anti-rRABV-GE sera with rRABV-GE and purified virus in ELISA. (**E**) Antibody titer of anti-rRABV-G-XVIII sera with rRABV-G-XVIII and purified virus in ELISA. Pre-immune mouse sera were used as a negative control. Endpoint titers are presented in square brackets. Pre-immune mouse sera were used as negative controls. Endpoint titers were determined as the highest serum dilution yielding an OD450 ≥ 0.1.

To investigate the immunogenicity of trimeric versus monomeric forms of the rabies virus glycoprotein, we immunized mice with recombinant rRABV-GE or rRABV-G-XVIII proteins formulated with or without adjuvant, following the schedule depicted in Figure 3A. An additional group received an inactivated rabies virus vaccine (CVS-11 strain) as a positive control.

To study the presence of antibodies recognizing rRABV-GE and rRABV-G-XVIII in polyclonal sera from mice immunized with inactivated RABV CVS-11 strain, immunoblotting was performed. The sera were assayed against purified rRABV-GE and rRABV-G-XVIII proteins. An intense band of about 51 kDa and 60 kDa appeared on the Western blot, indicating positive detection for rRABV-GE and rRABV-G-XVIII antibodies (Figure 3B). No band was noted in the pre-immune sera from the control group. These results showed that both rRABV-GE and rRABV-G-XVIII are antigenically similar to the native viral RABV-G, as evidenced by strong immunoreactivity against both recombinant proteins (Figure 3B).

Serum samples collected on Day 14 post-boost were analyzed by indirect ELISA to determine the magnitude and specificity of the antibody responses. When coated with purified rabies virus, sera from the inactivated rabies virus vaccine group showed the highest titers, as expected. Notably, sera from mice immunized with the inactivated rabies virus vaccine displayed significantly higher reactivity to the rRABV-G-XVIII compared to rRABV-GE (Figure 3C). Endpoint analysis confirmed that vaccine and rRABV-G-XVIII groups reached titers of up to 1:64,000, whereas the rRABV-GE group did not exceed 1:8000 (OD450 ≥ 0.1 cutoff), consistent with the weaker response of the monomeric construct.

To further explore antigen specificity and cross-reactivity, we evaluated ELISA binding of the antisera to both recombinant proteins and purified virus (Figure 3D,E). Sera from mice immunized with rRABV-GE showed increased reactivity to the homologous antigen when the adjuvant was used, but only limited cross-reactivity to the viral antigen. Furthermore, the sera showed a slight increase upon adjuvant inclusion. Endpoint titers reached as high as 1:128,000 against rRABV-GE, but responses to the viral antigen remained low (≤1:4000), highlighting the restricted specificity of the monomeric form. This suggests that the monomeric rRABV-GE may present non-native or less immunodominant epitopes.

In contrast, rRABV-G-XVIII-immunized sera exhibited high reactivity to rRABV-G-XVIII itself and demonstrated strong cross-recognition of the native viral antigen. Importantly, the inclusion of the adjuvant had minimal impact on the anti-viral titers elicited by rRABV-G-XVIII, which remained high regardless of adjuvant formulation (Figure 3E). Endpoint titers in this group consistently reached 1:128,000 against both recombinant and viral antigens, underscoring the strong mimicry of native conformational epitopes by the trimeric glycoprotein. This suggests that the trimeric form of the glycoprotein presents conformational epitopes that are closely aligned with those on the native viral particle, thereby driving a more relevant and functional immune response.

Taken together, these results underscore the immunological advantage of trimerizing the rabies glycoprotein via a collagen XVIII domain. The rRABV-G-XVIII construct induced a robust and broad humoral response that strongly recognized the native viral antigen, even in the absence of an adjuvant, highlighting its promise as a next-generation subunit vaccine candidate.

**Figure 4:**
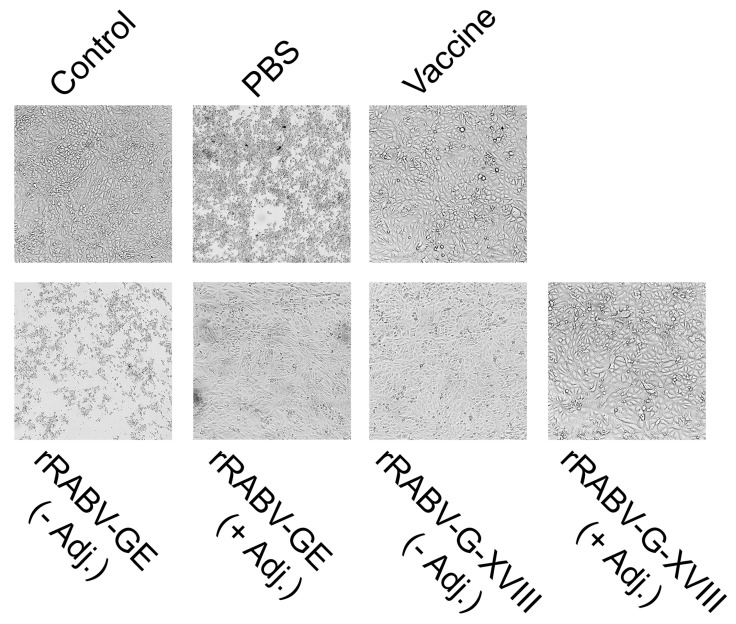
Representative images of the virus-neutralizing properties of antiviral sera from intramuscularly immunized rRABV-GE and rRABV-G-XVIII in Vero cell culture at 1:64 serum dilution. Microscopic images (×200) of Vero cells following the in vitro virus neutralization assay with rabies virus and immune sera. Reference and test sera were preincubated with 100 TCID_50_ of rabies virus for 2 h at 37 °C before inoculation onto monolayers. Serum from vaccinated mice was used as a positive control and serum from mice that received PBS was used as a negative control. The cytopathic effect (CPE) was assessed by light microscopy.

For the evaluation of viral neutralization capacity, sera from rRABV-GE- and rRABV-G-XVIII-immunized mice were tested in a cytopathic effect (CPE)-based neutralization assay. To test for their ability to prevent cytopathic effects of RABV CVS-11, the sera were mixed with an equal volume of 100 TCID_50_ RABV CVS-11 strain and incubated at 37 °C for 2 h. The resulting mixtures were assayed on Vero cells seeded in a 96-well plate. The cytopathic effect was monitored daily. The first signs of CPE were visible at approximately 72 h post-infection and became progressively more pronounced thereafter. The final evaluation for neutralization titers was performed at 6 days post-infection, when complete CPE was observed in the virus control wells. Neutralization titers were determined as the highest dilutions that resulted in a 100% inhibition of CPE. Representative images of the CPE and titer profiles of neutralizing antibody against CVS-11 are shown in Figure 4 and Table 1.

The antiserum, which was collected from the inactivated rabies virus (CVS-11 strain) immunized mice, served as a positive control, and it exhibited a neutralization titer of 1:512. The antisera obtained from the mice immunized with RABV-GE and rRABV-G-XVIII showed the neutralizing activities against the CVS-11 strain. Similarly to the results of the ELISA study, the neutralization effect was similar in the G-XVIII groups with and without adjuvant (1:512). The rRABV-GE resulted in a neutralization titer less than 1:32. A better neutralization effect at a titer of 1:64 was exhibited by the antiserum raised against rRABV-GE when the adjuvant was used, which corroborated the previous ELISA findings. These data provide additional evidence that rRABV-G-XVIII is a promising RABV CVS-11 vaccine candidate.

### 3.3. Protective Efficacy of Candidate Vaccines

**Figure 5:**
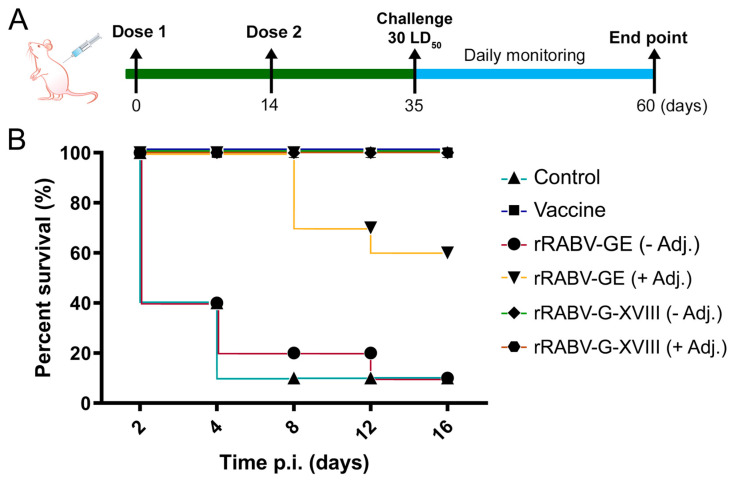
Survival of immunized mice after lethal rabies virus challenge. (**A**) Experimental design. BALB/c mice were immunized intramuscularly on day 0 (Dose 1) and day 14 (Dose 2) with the rRABV-GE (30 µg) and rRABV-G-XVIII (30 µg) without and with adjuvant. On day 35, all groups, including a positive (vaccine) and negative (PBS) control groups, were challenged intracerebrally with 30 LD_50_ of CVS-27 virus. Animals were monitored daily for 25 days post infection until the study endpoint at day 60. (**B**) Survival curves of immunized and control groups. Positive (vaccine) and negative (PBS) control groups are depicted in both panels for comparative purposes.

We also investigated the in vivo protection efficacy of the rRABV-GE and rRABV-G-XVIII vaccines against lethal rabies virus challenge (Figure 5). All control animals, which were immunized with PBS alone, died from rabies infection between 5 and 8 days after receiving the intracerebral CVS-27 virus challenge. The mice that succumbed to rabies displayed typical symptoms such as disheveled fur, hunched posture, lethargy, and hind-leg paralysis. The survival of immunized mice after this lethal challenge correlated closely with the levels of antibodies induced and the neutralization capability assessed by CPE. Initially, only 20% of mice immunized with RABV-GE survived the lethal challenge. However, incorporating Freund’s adjuvant into the vaccine formulation significantly increased survival rates within this group to 60% (Figure 5). The protein rRABV-G-XVIII, whether administered with or without adjuvant, provided complete protection (100%) against the lethal challenge. As anticipated, the inactivated rabies virus vaccine (CVS-11 strain) used as a control in this study also ensured 100% protection of immunized mice.

## 4. Discussion

In the present study, we used a novel approach to produce recombinant vaccine candidates against rabies. We selected the G protein of RABV that is known to be the main target for neutralizing antibodies, making it a critical component of rabies vaccines [26]. Furthermore, we expressed it either in an *E. coli* as rabies virus glycoprotein ectodomain (rRABV-GE) or in its more native trimeric form (rRABV-G-XVIII) to increase immunogenicity. We demonstrated that the trimerization of the rabies virus glycoprotein ectodomain (RABV-GE) through fusion with a human collagen XVIII trimerization domain significantly enhances the protein’s immunogenicity and protective efficacy.

Many therapeutic proteins and protein subunit vaccines contain heterologous trimerization domains, such as the widely used GCN4-based isoleucine zipper and the T4 bacteriophage fibritin foldon trimerization domains [27,28,29,30]. It has been shown that protein fragments that promote trimer formation, in particular the GCN4-based isoleucine zipper and foldon, can elicit a strong immune response when used in vaccine development [31]. A strong immune response against components of a vaccine, such as the trimerization domains, can be problematic because it may divert the immune system’s attention away from the actual target antigen (like a virus protein). This can reduce the vaccine’s overall efficacy by generating antibodies primarily against the scaffold rather than the intended pathogen. Additionally, an unwanted immune response against the carrier or scaffold might lead to adverse effects, such as immune complications or decreased tolerance upon repeated vaccinations [31]. The choice of the human collagen domain as a stabilizing element is due to its low immunogenicity and biocompatibility. Unlike frequently used bacterial or viral trimerization domains, the human collagen XVIII domain is less likely to elicit an immune response against the stabilizing element itself, which is especially important for use in vaccines.

The successful refolding and purification of recombinant RABV-G constructs expressed in *E. coli* demonstrate the feasibility of recovering functional glycoprotein domains from insoluble inclusion bodies. Notably, the engineered construct rRABV-G-XVIII, which includes a C-terminal trimerization domain derived from collagen XVIII, consistently formed soluble, trimeric assemblies post-refolding. In contrast, the monomeric ectodomain construct rRABV-GE, despite being solubilized and refolded under identical conditions, showed a strong tendency toward aggregation when analyzed by size-exclusion chromatography (Figure 2).

This difference in behavior underscores a critical insight: the absence of a trimerization scaffold likely renders the monomeric ectodomain structurally unstable in solution. In its native context, RABV-G is embedded in the viral envelope and stabilized by membrane anchoring and trimeric interactions [32]. Removal of the transmembrane and cytosolic domains, while improving solubility, also eliminates these structural supports. Consequently, the rRABV-GE construct may expose hydrophobic surfaces or adopt non-native conformations during the refolding process, predisposing it to aggregation.

Our results are consistent with previous studies showing that the native rabies glycoprotein exists as a trimer on the viral surface and that its proper quaternary conformation is critical for the presentation of conformational neutralizing epitopes [33,34]. The failure of monomeric forms, such as rRABV-GE, to elicit strong neutralizing responses further underscores the importance of structural mimicry of the native antigen. In our experiments, rRABV-GE without adjuvant elicited weak antibody responses and provided limited protection (20%), while the addition of the adjuvant improved survival to 60%. In contrast, rRABV-G-XVIII alone was sufficient to induce neutralization titers comparable to the inactivated rabies vaccine and conferred 100% protection, even without adjuvant, highlighting the immunological advantage of the trimeric configuration (Figure 5).

Recent advances in rabies vaccine research support the trend toward structure-guided design, particularly the use of trimer-stabilized or prefusion-stabilized glycoproteins to enhance immunogenicity. For example, studies using matrix-adjuvanted trimeric glycoprotein subunit vaccines or virus-like particles encoding multimerized glycoprotein domains have shown promising results in small animal models [35]. mRNA-based vaccines encoding pre-fusion-stabilized RABV-G also represent a promising platform, although proper expression and antigen processing remain critical challenges [36]. Our findings contribute to this growing body of evidence by showing that structural engineering using a human-derived trimerization domain can produce soluble, highly immunogenic rabies antigens using bacterial expression systems, which are cost-effective and scalable.

Importantly, the robust immune responses elicited by rRABV-G-XVIII in the absence of adjuvant suggest that the trimeric antigen itself provides sufficient immunostimulatory cues. This has implications for the development of simplified vaccine formulations, particularly for use in low-resource settings where cold-chain requirements and adjuvant safety profiles may pose challenges. Moreover, our approach allows for rapid production and purification under denaturing conditions, followed by effective refolding, yielding a protein product with high purity and preserved antigenicity.

In summary, our data indicate that trimerization of the rabies virus glycoprotein ectodomain substantially improves its immunogenic properties and protective capacity. The rRABV-G-XVIII construct represents a promising candidate for next-generation rabies subunit vaccines and could be further evaluated in combination with other delivery platforms, including nanoparticle or mRNA-based systems. Further studies will focus on assessing the duration of protection, cellular immune response and the efficacy of peripheral challenge models to more accurately reflect natural routes of rabies transmission.

## 5. Conclusions

This study highlights the advantages of trimerizing the rabies virus glycoprotein ectodomain using a human collagen XVIII trimerization domain to enhance its structural integrity, immunogenicity, and protective efficacy. The recombinant trimeric construct, rRABV-G-XVIII, induced strong virus-specific antibody responses and complete protection in mice against a lethal rabies challenge, even in the absence of adjuvant. These findings underscore the importance of preserving the native-like antigen structure in subunit vaccine design and position rRABV-G-XVIII as a promising candidate for the development of next-generation rabies vaccines.

## Figures and Tables

**Table 1:** Titer profiles of neutralizing antibody against CVS-11.

**Immunogen**	**Neutralization Titers**
Vaccine serum	1:512
rRABV-GE (− Adjuvant)	≤1:32
rRABV-GE (+ Adjuvant)	1:64
rRABV-G-XVIII (− Adjuvant)	1:512
rRABV-G-XVIII (+ Adjuvant)	1:512

## Data Availability

The original contributions presented in this study are included in the article/Supplementary Materials. Further inquiries can be directed to the corresponding author.

## Supplementary Materials

The following supporting information can be downloaded at https://www.mdpi.com/article/10.3390/vaccines13090971/s1. Figure S1: Full, uncropped Western blot analysis corresponding to Figure 1 and Figure 3 in the main text. Left panel: detection with polyclonal sera from the inactivated rabies virus. Right panel: detection with anti-His tag antibodies. Lane M: PageRuler™ Plus Prestained Protein Ladder, 10 to 250 kDa; lane 1: ectodomain (rRABV-GE); lane 2: rabies virus glycoprotein trimer (rRABV-G-XVIII); Figure S2: Original, uncropped Western blot membrane corresponding to Figure 1 and Figure 3 in the main text. Ponceau S staining of the PVDF membrane showing total protein load. Full immunoblot image with molecular weight markers indicated. Left panel: detection with polyclonal sera from the inactivated rabies virus. Right panel: detection with anti-His tag antibodies. Lane M: PageRuler™ Plus Prestained Protein Ladder, 10 to 250 kDa; lane 1: ectodomain (rRABV-GE); lane 2: rabies virus glycoprotein trimer (rRABV-G-XVIII); Figure S3: Original, uncropped gel images of agarose gel electrophoresis of PCR products. (A) PCR amplification of the rRABV-GE gene (1343 bp). (B) PCR amplification of the rRABV-G-XVIII gene (1612 bp). Lane M: GeneRuler 1 kb DNA Ladder; lanes 1–4: independent PCR products; Table S1. Densitometry results of Western blots.
