# Supplementary material for: Immunogenicity and Antigenicity of the Recombinant Ectodomain of Rabies Virus Glycoprotein Containing the Human Collagen XVIII Trimerization Domain
Source: Vaccines (Basel). 2025 Sep 12;13(9):971. doi: 10.3390/vaccines13090971 (PMC12474313; doi:10.3390/vaccines13090971)
Supplement: Supplementary file 1 [file vaccines-13-00971-s001.zip › vaccines-3826310-supplementary.pdf]

Supplementary Materials

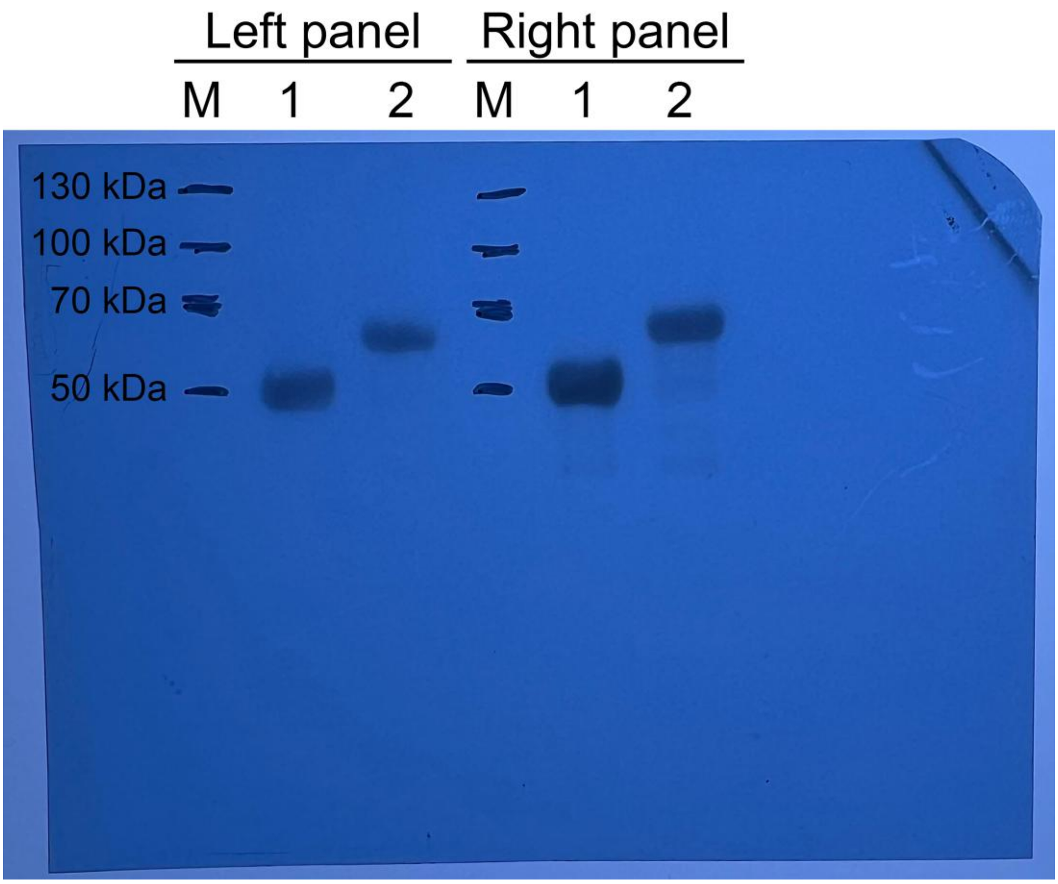

Figure S1. Full, uncropped Western blot analysis corresponding to Figures 1 and 3 in the main text. Left panel: detection with polyclonal sera from the inactivated rabies virus. Right panel: detection with anti-His tag antibodies. Lane M: PageRuler™ Plus Prestained Protein Ladder, 10 to 250 kDa; lane 1: ectodomain (rRABV-GE); lane 2: rabies virus glycoprotein trimer (rRABV-G-XVIII)

Table S1. Densitometry results of Western blots.

Sample	Lane/Panel	Integrated Intensity	Normalized (%)
rRABV-GE (51 kDa)	Left panel, Lane 1	11301530	100.0
rRABV-G-XVIII (60 kDa)	Left panel, Lane 2	11010979	97.4
rRABV-GE (51 kDa)	Right panel, Lane 1	10540051	93.3
rRABV-G-XVIII (60 kDa)	Right panel, Lane 2	10463480	92.6

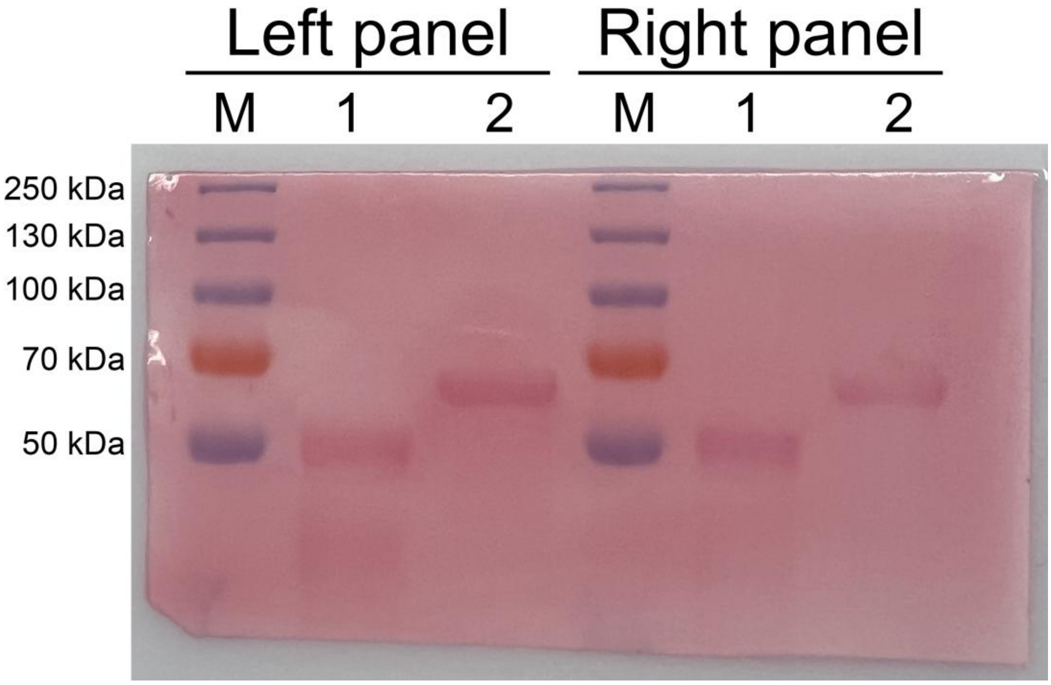

Figure S2. Original, uncropped Western blot membrane corresponding to Figures 1 and 3 in the main text. Ponceau S staining of the PVDF membrane showing total protein load. Full immunoblot image with molecular weight markers indicated. Left panel: detection with polyclonal sera from the inactivated rabies virus. Right panel: detection with anti-His tag antibodies. Lane M: PageRuler™ Plus Prestained Protein Ladder, 10 to 250 kDa; lane 1: ectodomain (rRABV-GE); lane 2: rabies virus glycoprotein trimer (rRABV-G-XVIII).

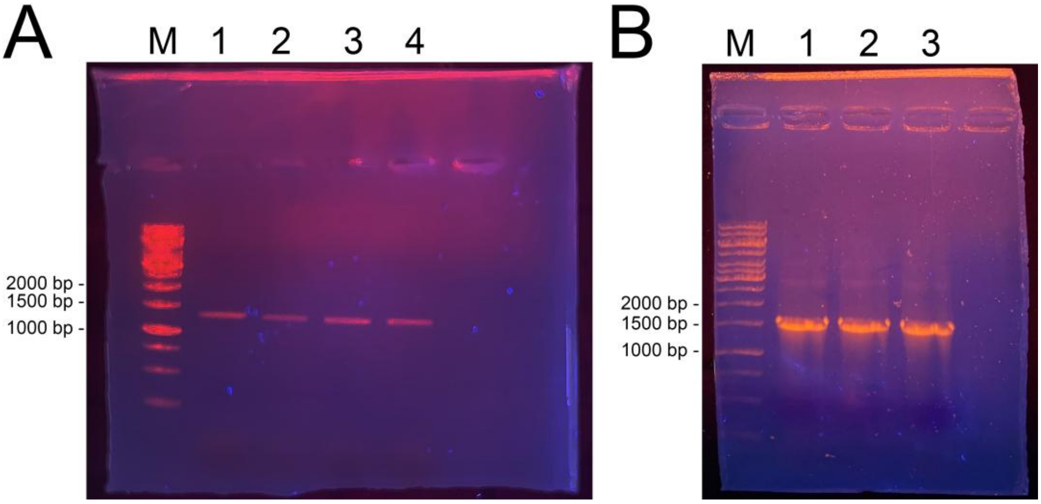

Figure S3. Original, uncropped gel images of agarose gel electrophoresis of PCR products. (A) PCR amplification of the rRABV-GE gene (1343 bp). (B) PCR amplification of the rRABV-G-XVIII gene (1612 bp). Lane M: GeneRuler 1 kb DNA Ladder; lanes 1–4: independent PCR products.
